# A Prognostic Risk Model of a Novel Oxidative Stress-Related Signature Predicts Clinical Prognosis and Demonstrates Immune Relevancy in Lung Adenocarcinoma

**DOI:** 10.1155/2022/2262014

**Published:** 2022-11-18

**Authors:** Xing Huang, Zhichao Lu, Min He, Yipeng Feng, Shaorong Yu, Bo Shen, Jianwei Lu, Pingping Wu, Banzhou Pan, Hanlin Ding, Chen Chen, Yidan Sun

**Affiliations:** ^1^Department of Pathology, Jiangsu Cancer Hospital & Jiangsu Institute of Cancer Research & Nanjing Medical University Affiliated Cancer Hospital, China; ^2^Research Center of Clinical Medicine, Affiliated Hospital of Nantong University, Nantong 226001, China; ^3^Department of Thoracic Surgery, Nanjing Medical University Affiliated Cancer Hospital & Jiangsu Cancer Hospital & Jiangsu Institute of Cancer Research, 21009 Nanjing, China; ^4^Jiangsu Key Laboratory of Molecular and Translational Cancer Research, Cancer Institute of Jiangsu Province, Nanjing, China; ^5^The Fourth Clinical College of Nanjing Medical University, Nanjing, China; ^6^Department of Oncology, Jiangsu Cancer Hospital & Jiangsu Institute of Cancer Research & The Affiliated Cancer Hospital of Nanjing Medical University, Nanjing 210000, China; ^7^The Comprehensive Cancer Centre of Nanjing Drum Tower Hospital, The Affiliated Hospital of Nanjing University Medical School, China; ^8^Department of Oncology, First Teaching Hospital of Tianjin University of Traditional Chinese Medicine, Tianjin, China

## Abstract

Lung adenocarcinoma (LUAD) is among the most prevalent malignant lung cancers with a poor prognosis due to high invasiveness and lethality despite multiple treatments. Since the lung is an important organ associated with oxidative stress, and it has been confirmed that oxidative stress represents a potential cancer-specific depletion, it is of important significance to investigate and evaluate the clinical value of oxidative stress mechanisms regulating tumor cell apoptosis. Furthermore, there are few studies on the impact of the microenvironment on reaction to immune-checkpoint inhibitors (ICIs) in patients with LUAD. Based on the TCGA-LUAD dataset, which is stratified into a training set as well as a validation set in a ratio of 2 : 1, this investigation constructs and validates a prognostic predictive power of a gene signature model of oxidative stress-related prognostic signatures. To ascertain the differences between the high-risk score group and the low-risk score group in tumor-infiltrating lymphocytes and patients' response to ICI therapy. This oxidative stress-related prognostic gene signature is composed of MAP3K19 and NTSR1 and is an independent prognosis-related factor in the LUAD group. The outcome of patients having a low risk score is better, and the difference was statistically significant, and individuals with a low risk score had a larger number of infiltrating immune cell distribution in the tumor microenvironment, which was closely related to clinical outcome. Our study suggests that the synergistic effect of oxidative stress-related prognostic gene markers-MAP3K19 and NTSR1 has clinical significance in the prognosis identification and immunotherapy of LUAD patients. Thus, the results may help to better intersect the oxidative stress-related mechanisms in clinical value in LUAD but requires prospective validation.

## 1. Introduction

Lung cancer is the major important cause of tumor-related mortalities worldwide [1, 2]. LUAD is the most significant subtype. Although many clinical studies have confirmed that multitargeted drugs and immunotherapy can prolong the overall survival (OS) and improve the objective response rate (ORR) of LUAD patients [3–5], the rapid progression of the disease due to multi-drug resistance is currently very difficult. Few targeted treatment options [6–9]. With the in-depth research in many aspects [10, 11], it is beneficial to discover small molecule inhibitors. Furthermore, significant literature confirmed the hypothesis that the tumor microenvironment (TME) enhances tumor growth through paracrine signaling [12]. So, more investigation should be taken to improve the outcome among LUAD patients.

Redox homeostasis is crucial in not only the survival of normal cells but also cancerous cells. Oxidative stress (OS) is predominantly triggered by an imbalance between cellular antioxidant mechanisms and metabolically generated oxidative free radical species [13, 14]. This imbalance eventually causes the excessive buildup of reactive oxygen species (ROS) within body cells, leading to irreversible or reversible injury to the body [15]. Nevertheless, numerous tumors have increased levels of ROS and exhibited signs of chronic oxidative stress as a result of oncogenic injury, hypoxia, metabolic malfunctions, and proteotoxic stress [13]. Increased ROS is hypothesized to enhance the progression of tumors at sublethal levels by inducing the mutations and changing cell signaling [16]. Nonetheless, to block excessive oxidative injury, tumors generally upregulate the antioxidant pathways [17]. Consequently, numerous cancerous cells are hypersensitive to perturbation of ROS levels. Excessive oxidative stress is known to aggravate the cytotoxic impacts of chemotherapy, and efforts are being made to enhance ROS generation in these environments [18, 19].

In this study, we attempted to obtain oxidative stress-related expression profiling data for LUAD from The Cancer Genome Atlas (TCGA) database and aggregated clinical information and transcriptomes from 445 patients in TCGA-LUAD with complete clinical informatics data and express the data, dividing it into a training set (2/3 of the total, *n* = 296) as well as a test set (1/3 of the total, *n* = 149). We then performed univariate Cox proportional hazards regression in both the training and the validation sets, respectively, to identify genes with prognostic values utilizing the expression data of 147 oxidative stress-related genes. Cox *p* values < 0.05 indicated a coexpression network of 35 oxidative stress DEGs associated with LUAD overall survival, with clinical information and transcriptomic expression data. Based on the LASSO algorithm, the prognosis-related gene signatures composed of 3 and 8 oxidative stress-related genes were screened in the training set and the test set, respectively. Therefore, we obtained a LUAD prognostic risk model composed of MAP3K19 and NTSR1 by intersection difference analysis. We further validated the subgroup prognostic risk model by Kaplan-Meier (KM) analysis as well as receiver-operating characteristic curve (ROC) analysis. Additionally, the link between the risk model and the TME was ascertained by both the ESTIMATE R software package and the CIBERSORT tool. Lastly, differentially infiltrating immune cells were discovered in the two risk groups, and MAP3K19 and NTSR1 were found to be statistically significant with immune-related genes. In conclusion, our study suggests that oxidative stress-related risk models may provide a viable prognostic tool and important function in the modulation of immune cell distribution in the LUAD tumor microenvironment. Our hypothesis is that this oxidative stress-related prognostic model gene signature has the capacity to anticipate the targeting and prognosis of LUAD. The aim of our investigation was to create and validate an oxidative stress-related LUAD prognostic model and to explore its predictive effect on poor prognosis in LUAD. Assess and validate prognostic power and its independent prognostic value. Our goal was to guide the clinical application of this oxidative stress-related prognostic model gene signature in LUAD.

## 2. Materials and Methods

**Figure 1:**
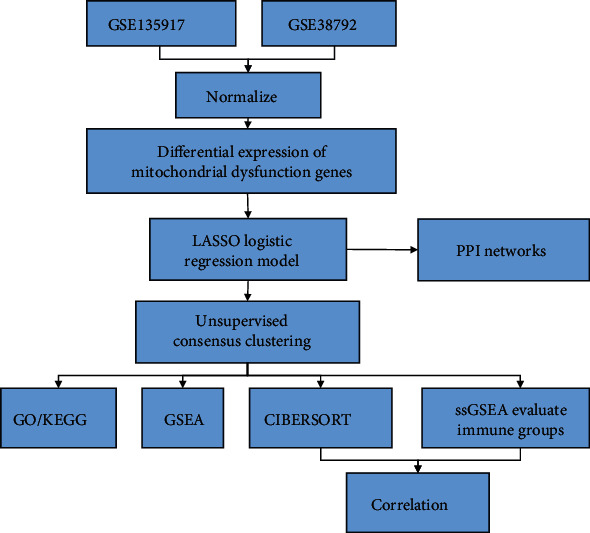
Flowchart for constructing and validating a prognostic model for TCGA-LUAD overall survival.

Figure 1 presents the flow chart for the bioinformatics analysis. R language software (Version 4.0.3) [20] accomplished all the statistical analyses, and *p* < 0.05 denoted a statistically significant difference without a designated setting.

### 2.1. Data Acquiring and Cleaning

Transcriptomic data (reads), as well as relevant clinical data encompassing survival status, age, sex, grade, and stage of LUAD patients, were retrieved from TCGA (https://portal.gdc.cancer.gov/) database. In total, 551 samples were incorporated in this investigation (54 normal lung samples and 497 LUAD samples). The initial expression data were normalized with the aid of the trim mean of *M* values (TMM) algorithm in the “limma” package [21], and genes with an average expression not exceeding 1 were excluded. The “limma” package was additionally utilized for MTG differential expression analysis. The criteria for identifying differentially expressed genes in our study were |logFC| > 1 and adj.*p* < 0.05. The read counts were transformed to TPM values, and a log2(*x* + 1) conversion was conducted for further analyses since the TPM values were identical to the microarray values.

We use the *k*-fold cross-validation method to divide the sample set into *k* mutually exclusive subsets of similar size, each of which keeps the data distribution as consistent as possible. Then, each time the union of *k* − 1 subsets is used as the training set, and the remaining subset is used as the test set, so that *k* sets of training/testing sets can be obtained. Perform *k* training and testing times, and return the average of the *k* test results.

### 2.2. Identification of Differentially Expressed Oxidative Stress-Related Genes with Prognostic Value in LUAD Tissue and Normal Lung Tissue

As per the search term “oxidative stress,” from the OMIM database, NCBI gene function module, and the GeneCard database, a total of 9469 human genes which are linked to oxidative stress were collected. Based on this, we intersected these oxidative stress-related genes with the differentially expressed genes in the TCGA-LUAD database to set and screen 147 oxidative stress-related genes with significant expression differences for subsequent analysis. We used a string-generated protein interaction (PPI) network database (version 11.0) of 147 oxidative stress-related genes differentially expressed in the TCGA-LUAD dataset to construct a molecular interaction network for analyzing closely interacting differential genes. Then, the PPI was exported, and the Cytoscape [22] software was used for further analysis, the network properties of each node were calculated, and the MCODE [23] and Cytohubba [24] were used to mine the hub nodes based on the degree of the nodes. The high level of linkage may have an extremely important function in the modulation of the whole biological process, which deserves further study.

### 2.3. Creation and Validation of a Prognostic Oxidative Stress-Related Signature for LUAD

We randomly divided 445 patients with complete clinical informatics data in TCGA-LUAD into a training set (2/3 of the total, *n* = 296) and a test set (1/3 of the total, *n* = 149). We then performed univariate Cox proportional hazards regression in the training set and validation set, respectively, to discover genes with prognostic value, utilizing the expression data of 147 DE-oxidative stress-related genes. A Cox *p* value < 0.05 connoted a substantial correlation with overall survival (OS). The degree of correlation and prognostic value were selected using the “Venn” R package for cross-analysis and genes affecting prognostic value. To avert overfitting, all genes with *p* values < 0.05 were subjected to a least absolute shrinkage and selection operator (LASSO) analysis using the package glmnet. LASSO regression is usually a regularization method for high-dimensional predictor selection. The hazard system used LASSO Cox proportional hazards to build a score-identifying gene signature to anticipate OS models for LUAD patients. Prediction scores are weighted sums using developed genes, with coefficient regularization by LASSO. After being filtered by the LASSO model, the selected genes are constructed by the multivariate Cox proportional hazards model to construct an immune-related risk model: risk score = level of gene *a*^∗^ coefficient *a* + level of gene *b*^∗^ coefficient *b* + level of gene *c*^∗^ coefficient *c* + ⋯+level of gene *n*^∗^ coefficient *n*. The risk score in the model represents the prognosis of LUAD patients; the smaller the risk score, the better the prognosis. Patients were categorized into two risk groups utilizing the median risk score that served as a cutoff value. The “pheatmap” R package used scatter diagrams to show how the risk scores and survival durations of all patients were distributed. The “stats” R package used principal component analysis (PCA) to measure the gene expression of an established signature. Kaplan-Meier (K-M) survival analysis and time-dependent ROC analysis based on OS were carried out utilizing the “survival” package, the “survminer” package [25], and the “timeROC” package [26] in R to measure the prognostic accuracy of the gene signature in the derivation set and validate it in the validation set. Kaplan-Meier survival curves were used to derive the predictive power, whereas a log-rank *p* value < 0.05 denoted a statistical significance (utilizing package survival and survminer). To ascertain predictive power of the immune signature's, time-dependent ROC curves (package survivalROC) were employed. Subsequently, logistic regression was used for correlation analysis of dichotomous clinical subgroup variables.

### 2.4. Pathway and Function Enrichment Analysis

To investigate the biological value of these differentially expressed IRGs, we utilized DAVID Bioinformatics Resources 6.8 to conduct a pathway and function enrichment analysis. The visualization procedure was carried out via the package “ggplot2” [25].

### 2.5. Gene Set Enrichment Analysis (GSEA) of Oxidative Stress Prognostic Model in LUAD

GSEA is a computational approach utilized to ascertain if a predefined set of genes exhibits statistical differences between two biological states, which is typically employed to obtain the expression in a dataset sample. Changes in the pathway as well as activity of biological processes were analyzed [23]. To investigate the variations in biological processes between the two groups of samples, utilizing the gene expression profile dataset, we retrieved the reference gene set “c2.cp.kegg.v7.4.entrez.gmt” from the MSigDB database, using the R package “clusterProfiler” The GSEA method is included in “enrichment analysis and visualization of datasets. *p* value < 0.05 was considered statistically significant.

Gene set variation analysis (GSVA) [27] is a nonparametric unsupervised analysis method, which is mainly used to evaluate the microarray by transforming the expression matrix of genes between various samples into the expression matrix of gene sets between samples. Transcriptome gene set enrichment findings assess if various metabolic pathways are enriched across various samples. To investigate the biological process variation occurring in the two groups of samples, we utilized the R package GSVA to carry out the GSVA procedure based on the gene expression profile dataset and downloaded the reference “c2.cp.kegg.v7.4. entrez” gene set from the MSigDB database to compute the enrichment score of every sample in each pathway in the dataset, combined with the R package limma to screen significantly different pathways, the GSVA enrichment results were visualized based on the heatmap utilizing the R package pheatmap, and *p* value < 0.05 denoted a statistically significant significance.

### 2.6. Establishment and Verification of Nomogram

Nomogram has been extensively utilized for anticipating cancer-related prognosis. This approach allows individualized approximates of the likelihood of recurrence, mortality, or drug adherence. Using the prognostic model, this investigation developed the nomogram in the TCGA-LUAD cohort by incorporating the above-stated clinical parameters to anticipate the OS probably over 1, 3, and 5 years. Furthermore, calibration curves evaluated the fitness between actual survival statuses with visualized survival status of the developed nomogram via bootstrap methods (1,000 replicates). The values of prognosis evaluation between risk signature, stage, and the nomogram were compared via ROC curves at 1 year, 3 years, and 5 years, correspondingly.

### 2.7. Computation of Immune Infiltration Score Was Premised on the Gene Groups Previously Determined

We downloaded the unified as well as the standardized pan-cancer dataset from the UCSC (https://xenabrowser.net/) database: TCGA TARGET GTEx (PANCAN, *N* = 19131, *G* = 60499). Additionally, we extracted gene expression data in every sample and further screened the sample sources: Primary Blood-Derived Cancer-Peripheral Blood (TCGA-LAML), Primary Tumor, Metastatic TCGA-SKCM, Primary Blood-Derived Cancer-Bone Marrow, Primary Solid Tumor, and Recurrent Blood-Derived Cancer-Bone Marrow samples, further log2(*x* + 0.001) transformation was carried out on every expression value; in addition, we also extracted the gene expression profile of every tumor, respectively, and mapped the expression profile. On the GeneSymbol, the R software package ESTIMATE (version 1.0.13, https://bioinformatics.mdanderson.org/public-software/estimate/, doi:10.1038/ncomms3612) [28] was further used to calculate the gene expression in each tumor, stromal, immune, and ESTIMATE scores for each patient.

### 2.8. GO, KEGG, and Immune Infiltration Enrichment Analyses for Risk-Related DEGs

In accordance with the risk grouping, normalized gene expression matrixes of the derivation set as well as the validation set generated above were employed with the “limma” R package to detect risk-related DEGs with the cut-off criteria of |logFC| ≥ 1 and adj.*p* < 0.05, correspondingly. Risk-related DEGs were analyzed with both GO and KEGG utilizing the “clusterProfiler” R package. Next, single-sample GSEA (ssGSEA) for immune infiltration was adopted with the “GSVA” R package to ascertain the infiltration score of immune cells and the immune-related roles.

### 2.9. Calculation of Immune Infiltration Score

CIBERSORT played a crucial function in calculating an absolute immune infiltrate score for the primary tumor samples. The default CIBERSORT parameters were instrumental in generating the curated CIBERSORT signature matrix. It roughly yielded the expected relative abundances. We performed a pan-cancer analysis of the CIBERSORT score, including the correlation of immune score in pan-cancer, analyzed the correlation of MAP3K19 and NTSR1 with the distribution of various immune cells for LUAD patients, and visualized them with heatmaps and scatter plots. The tumor purity, as well as stromal scores, were determined utilizing the Estimation of Stromal and Immune Scores ESTIMATE based on RNA-seq data and global proteomic data.

### 2.10. Quantitative Reverse Transcription-Polymerase Chain Reaction (qRT-PCR)

Total RNA extracted from lung adenocarcinoma tissue and para-cancerous tissue with Trizol Reagent (Invitrogen, USA) were reverse transcribed with HiScript III 1st Strand cDNA Synthesis Kit (Vzayme, China). Next, HiScript II One Step RT-PCR Kit (Vzayme, China) and qRT-PCR analysis were used for detecting cDNA expression levels, and GAPDH was used as internal reference. Primers were shown as follows: GAPDH, forward (F): 5′-AATGGGCAGCCGTTAGGAAA-3′, reverse(R): 5′-GCCCAATACGACCAAATCAGAG-3′; NTR1, forward (F): 5′-TCATCGCCTTTGTGGTCTGCT-3′, reverse (R): 5′-TGGTTGCTGGACACGCTGTCG-3′; MAP3K19, forward (F): 5′-AGGAGTTCGACCAAGATGGTG-3′, reverse (R): 5′-GGTCGAAAACTCTTCTGTCCTG-3′.

### 2.11. Immunohistochemistry (IHC) Staining

After deparaffinization and dehydrating the tissue sections, they were subjected to epitope retrieval, treated with H_2_O_2_, and blocked against nonspecific bindings. The tissues were then incubated overnight with anti-NTSR1 antibodies (1 : 100, Abcam, ab217134) and anti-MAP3K19 antibodies (1 : 100, Invitrogen, PA5-29285) at 4°C. Subsequently, the tissue sections were incubated with secondary antibodies (1 : 1000, Proteintech, SA00001-2) for two hours at ambient temperature. The signal was detected with an enhanced DAB staining kit (Proteintech, China).

### 2.12. Statistical Analysis

The Spearman correlation test was performed to investigate the link between two variables that were nonlinearly linked. The Student's *t*-test, on the other hand, was utilized in comparing the normally distributed data, whereas the chi-square test was carried out to contrast pairwise and categorical features in various subgroup. Univariate as well as multivariate Cox regression analyses assessed the influences of the immune signature and numerous clinic-pathological parameters on the survival of patients. The package pheatmap was vital in plotting heatmaps. A two-sided *p* < 0.05 was statistically significant. Kruskal-Wallis test was employed for one independent variable with two or more levels and an ordinal dependent variable. K-M analysis measured the proportion of individuals living for a particular period, whereas the log-rank test evaluated the significance of differences. In these investigations, statistical analysis was performed by R software (4.0.0). A two-tailed *p* value of <0.05 was statistically significant unless otherwise stated.

## 3. Result

### 3.1. The Characteristics of Patients

RNA-sequencing profiles of a total of 445 LUAD sample as well as clinic-pathological data were obtained from the UCSC Xena TCGA-LUAD dataset. Then, randomization was conducted to divide them into two groups, namely, the training set (331 patients) and the test set (166 patients) (Table 1).

### 3.2. Prognosis-Related Differential Oxidative Stress Gene Signatures Are Independent Prognostic Factors for LUAD Patients

**Figure 2:**
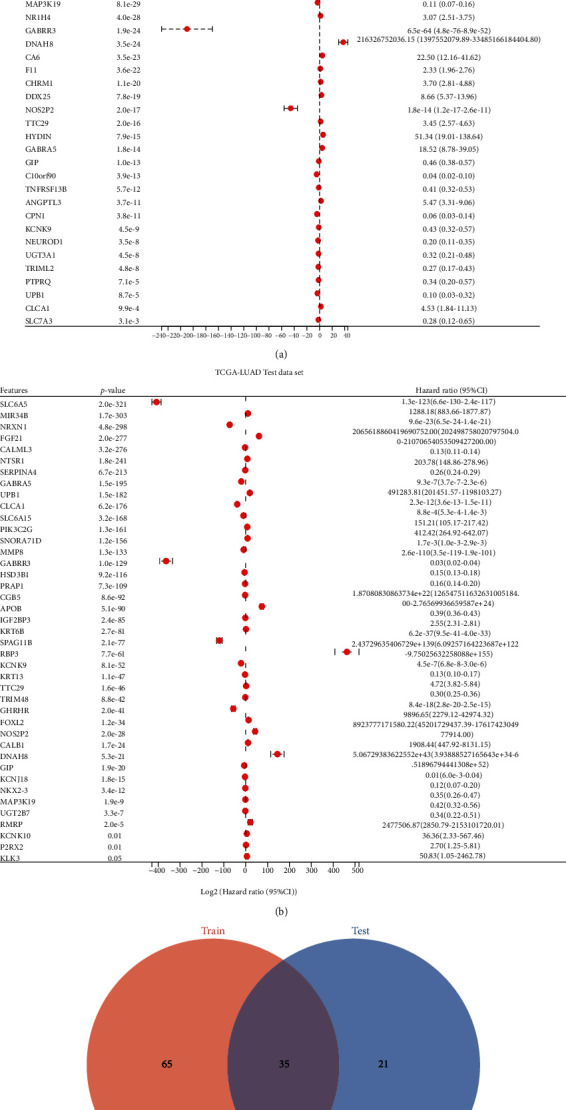
Univariate Cox analysis based on TCGA-LUAD internal training set and validation set and screening of differentially oxidative stress genes associated with LUAD prognosis. (a) Forest plot displays the findings of univariate Cox analysis of differential oxidative stress genes associated with LUAD prognosis in the training set; (b) forest plot demonstrates the findings of univariate Cox analysis of differential oxidative stress genes associated with LUAD prognosis in the training set; (c) Venn shows 35 prognosis-related differential oxidative stress genes at the intersection of the two sets.

To determine the prognostic value of differentially oxidative stress genes, we did a univariate Cox regression analysis. There were 100 and 56 xenooxidative stress genes significantly linked to the overall survival (OS) in the training and validation sets, respectively (Figures 2(a) and 2(b)). A total of 35 differentially oxidative stress genes associated with prognosis were obtained by intersecting them (Figure 2(c)).

### 3.3. Construction of PPI Network of DEGs Related to Oxidative Stress

**Figure 3:**
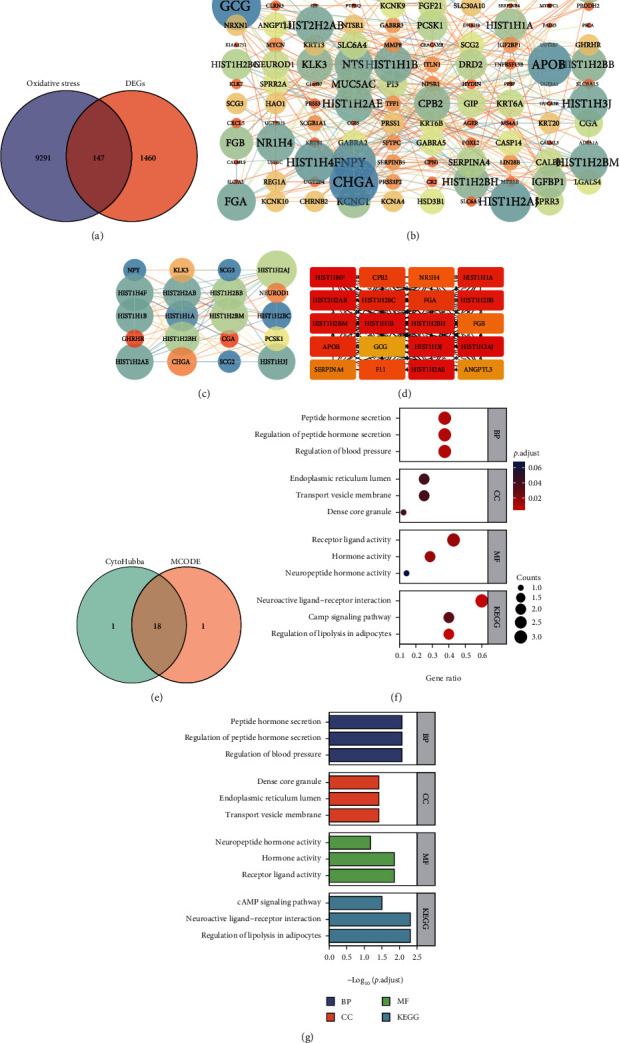
PPI network construction of differentially expressed genes related to oxidative stress and enrichment analysis of hub genes. (a) 1607 differentially expressed genes (|LogFC| > 2, adj. *p* value < 0.05) between normal samples and LUAD samples in the TCGA-LUAD dataset, and 9469 oxidative stress genes were intersected, and 147 differentially expressed genes were obtained oxidative stress genes; (b) the Network Analyzer tool of Cytoscape (v3.7.2) visualizes the PPIs of 147 differentially expressed oxidative stress genes with the largest confidence interaction score of 0.4. As the degree of interaction increases, the color gradually changes from yellow to blue, and the font changes from small to large; (c) the MCODE plug-in screened and visualized the closely related genes of the PPI network module; (d) the CytoHubba plug-in was used to screen the top 20 closely related genes; (e) the intersection of the two methods is shown by Venn diagram to obtain 18 closely related differentially oxidative stress-related genes; (f, g) based on 18 Hub Gene Ontology (GO) enrichment analysis of genes as well as KEGG pathway enrichment bubble plots and histograms.

We first performed a differential analysis between 59 normal samples and 535 LUAD samples in the TCGA-LUAD dataset using the limma algorithm and obtained a total of 1607 differentially expressed genes (|logFC| > 2, adj. *p* value < 0.05). Based on this differential result, 9469 previously identified oxidative stress genes were intersected, and 147 differentially expressed oxidative stress genes were obtained (Figure 3(a)). The STRING database constructed a protein-protein interaction network (PPI) to reflect the intermolecular interactions, and the largest confidence interaction score was established at 0.4, which was analyzed and visualized by the Network Analyzer tool of Cytoscape (v3.7.2) (Figure 3(b)). PPI network modules were screened for closely linked genes using the MCODE plugin and visualized (Figure 3(c)). At the same time, the CytoHubba plugin was used to screen the Top20 closely linked genes (Figure 3(d)). The intersection of the two approaches was shown by the Venn diagram, obtaining 18 closely related differentially oxidative stress-related genes (Figure 3(e)), including HIST2H2AB, HIST1H2BC, SCG3, HIST1H2AJ, CHGA, HIST1H3J, HIST1H1B, HIST1H2BM, HIST1H1A, SCG2, HIST1H4F, HIST1H2AE, GHRHR, KLK3, NEUROD1, NPY, HIST1H2BB, HIST1H2BH, and CGA.

### 3.4. Functional Enrichment Analysis

We analyzed these 18 closely related differentially oxidative stress-related genes. Gene Ontology (GO) enrichment analysis employing the above genes suggests that these hub genes exist in the membrane of transport vesicles, endoplasmic reticulum lumen, mast cell granules, and transport vesicles, which can affect blood pressure, hormone secretion, and G protein. Coupling cyclic, coupling receptor signaling pathway, nucleotide second messenger, and other functions play a regulatory role and have certain applications for receptor-ligand activity, neuropeptide hormone activity, neuropeptide receptor binding, and chemotactic activity value. The enrichment of BP set was mostly concentrated in oxidative phosphorylation, mitochondrial translation elongation, mitochondrial translation termination, translation termination, and purine ribonucleoside triphosphate metabolism. CC enrichment was mostly concentrated in the inner mitochondrial membrane, mitochondrial protein complexes, mitochondrial matrix, organelle ribosomes, and mitochondrial ribosomes. The enrichment of MF was mostly concentrated in the structural components of ribosomes, proton transmembrane, transporter activity, electron transfer activity, cytochrome-c oxidase activity, and heme-copper terminal oxidase activity. This suggests that the Hub gene may be related to the transmembrane transport of cells in terms of molecular structure. Combined with all the above enrichment results, we speculate that key genes related to oxidative stress may play a role in the interaction process and related mechanisms between cells. The KEGG pathway enrichment results indicated that differential oxidative stress hub genes were enriched in modulation of lipolysis in adipocytes, neuroactive ligand-receptor interaction, cAMP signaling pathway, maturity onset diabetes of the young, ovarian steroidogenesis, and other pathways. This result suggests that the differentially expressed oxidative stress-related genes can affect the occurrence and progression of LUAD via the above potential biological functions and molecular pathways (Figures 3(f) and 3(g) and Table 2).

### 3.5. Establishment and Validation of Prognostic Gene Signature Associated with Oxidative Stress in TCGA-LUAD

**Figure 4:**
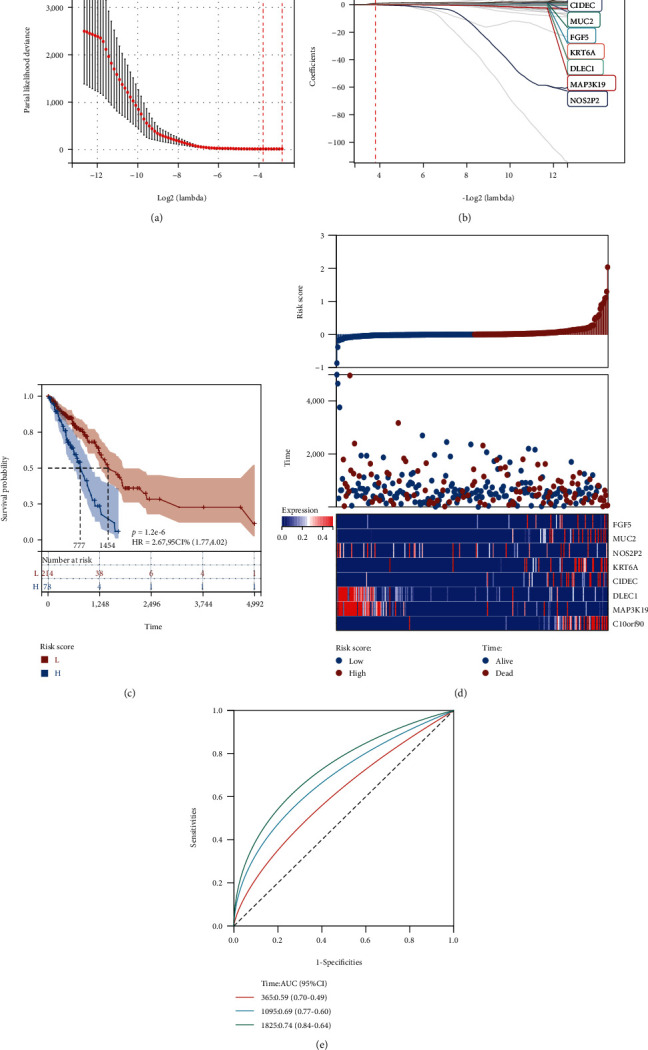
LASSO analysis of 147 differentially oxidative stress genes revealed the distribution and prognostic analysis of 8 gene markers. (a) Cross-validation of tuning parameter selection in the LASSO model; (b) LASSO coefficient spectrum of 8 prognostic differentially oxidative stress-related genes; (c) OS-based K-M survival of patients in the two risk groups in the TCGA-LUAD training set curves; (d) risk analysis graph of 8 prognostic differential oxidative stress gene signatures; the upper panel shows the distribution as well as the median of risk scores in the TCGA-LUAD training set; the middle panel shows the distribution of patients in various risk groups; the lower panel heatmap for the differential expression of 8 genes in the two risk groups; (e) the prognostic performance of risk scores in the TCGA-LUAD training set at 1, 3, and 5 years is validated utilizing AUC of time-dependent ROC curves.

To avert overfitting, we additionally performed a LASSO-Cox analysis. In order to avoid the influence of confounding factors, we first performed LASSO-Cox analysis on 147 differentially oxidative stress genes in the training set and established 8 gene signatures, including C10orf90, CIDEC, MUC2, FGF5, KRT6A, DLEC1, MAP3K19, and NOS2P2. Patients were categorized into high- and low-risk groups as per the median risk score with each group containing 167 participants. To tune parameter selection through the LASSO model, we utilized cross-validation (Figure 4(a)). The coefficient profiles of LASSO for the eight prognostic differentially oxidative stress-related genes were shown (Figure 4(b)). At the same time, we show eight prognostic differential oxidative stress gene signature risk analysis graphs (Figure 4(d)). The upper graph shows the distribution as well as the median of risk scores in the training set of TCGA-LUAD, and the middle graph shows the distribution of individuals in various risk groups. The figure below is a heatmap of the differential expression of 8 genes in the two risk groups. Further assessment of the gene signatures' prognostic value as well as the predictive performance utilizing both K-M survival and time-dependent ROC analyses was done, both of which yielded remarkable results. To account for survival outcomes, we observed a statistically significantly higher number of dead participants in the high-risk group in contrast with the low-risk group (*p* = 1.2*e* − 6, HR = 2.67 (1.77, 4.02)) (Figure 4(c)). The AUC reached 0.59 (0.70-0.49) at 1 year, 0.69 (0.77-0.60) at 3 years, and 0.74 (0.84-0.64) at 5 years (Figure 4(e)).

**Figure 5:**
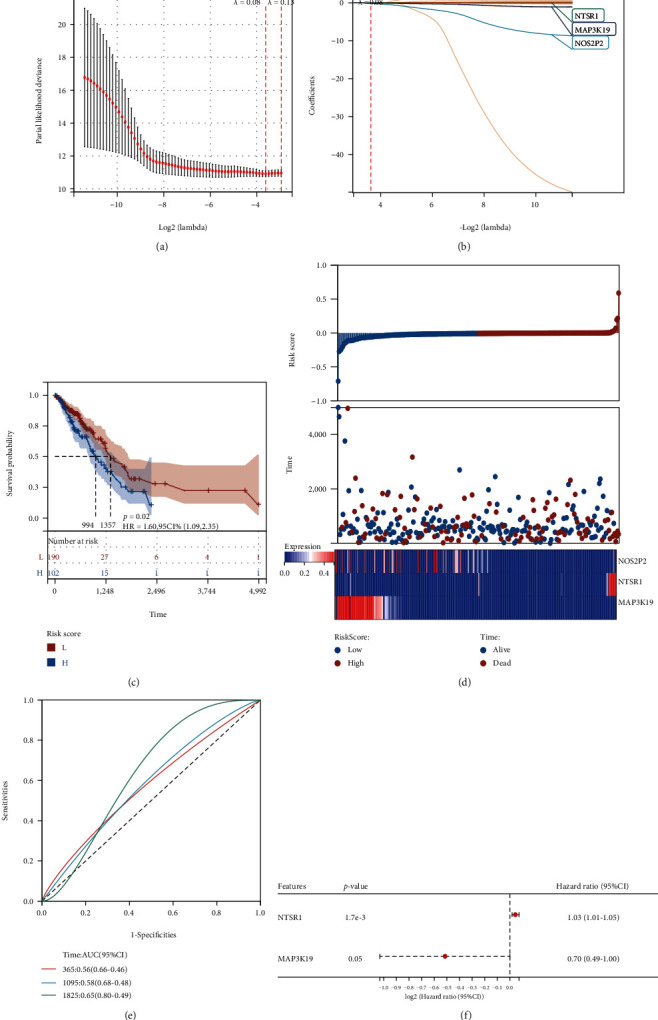
LASSO analysis of 35 differentially oxidative stress genes in the TCGA-LUAD training set showing the distribution and prognostic analysis of 3 gene signatures. (a) Cross-validation of tuning parameter selection in the LASSO model; (b) LASSO coefficient spectrum of 3 prognostic differentially oxidative stress-related genes; (c) OS-based K-M survival of patients in the two risk groups in the TCGA-LUAD training set curves; (d) 3 prognostic differential oxidative stress gene signature risk analysis graphs; the upper graph shows the distribution as well as the median of risk scores in the TCGA-LUAD training set; the middle graph shows the distribution of individuals in various risk groups, and the lower graph shows heatmap of differential expression of 3 genes in the two risk groups; (e) the prognostic performance of risk scores in the TCGA-LUAD training set over 1, 3, and 5 years is validated utilizing AUC of time-dependent ROC curves; (f) based on the training set, multivariate Cox proportional hazards regression forest plot for a 3-gene prognostic model.

Furthermore, LASSO-Cox analysis was conducted on the above 35 prognosis-related differential oxidative stress genes in the training set and validation set, correspondingly. A 3-gene prognostic model was predicted among the genes in the training set, including NTSR1, MAP3K19, and NOS2P2 as predictors of patient prognosis (Figures 5(a) and 5(b)). Not only K-M survival but also time-dependent ROC analysis findings were significant. The high-risk group had a greater mortality risk in contrast with the low-risk group (*p* = 0.02, HR = 1.60 (1.09, 2.35)) (Figure 5(c)). We show 3 prognostic differential oxidative stress gene signature risk analysis graphs, the upper graph shows the distribution and median of risk scores in the TCGA-LUAD training set, and the heatmap reflects the expression differences of model genes (Figure 5(d)). The AUC reached 0.56 (0.66-0.46) at 1 year, 0.58 (0.68-0.48) at 3 years, and 0.65 (0.80-0.49) at 5 years (Figure 5(e)). In the multivariate Cox regression analysis, NTSR1, MAP3K19, and NOS2P2 were incorporated. Multivariate Cox proportional hazards regression based on the training set suggested that only NTSR1 and MAP3K19 were jointly used as predictors of poor prognosis at high risk of LUAD, with significant statistical significance (Figure 5(f)).

**Figure 6:**
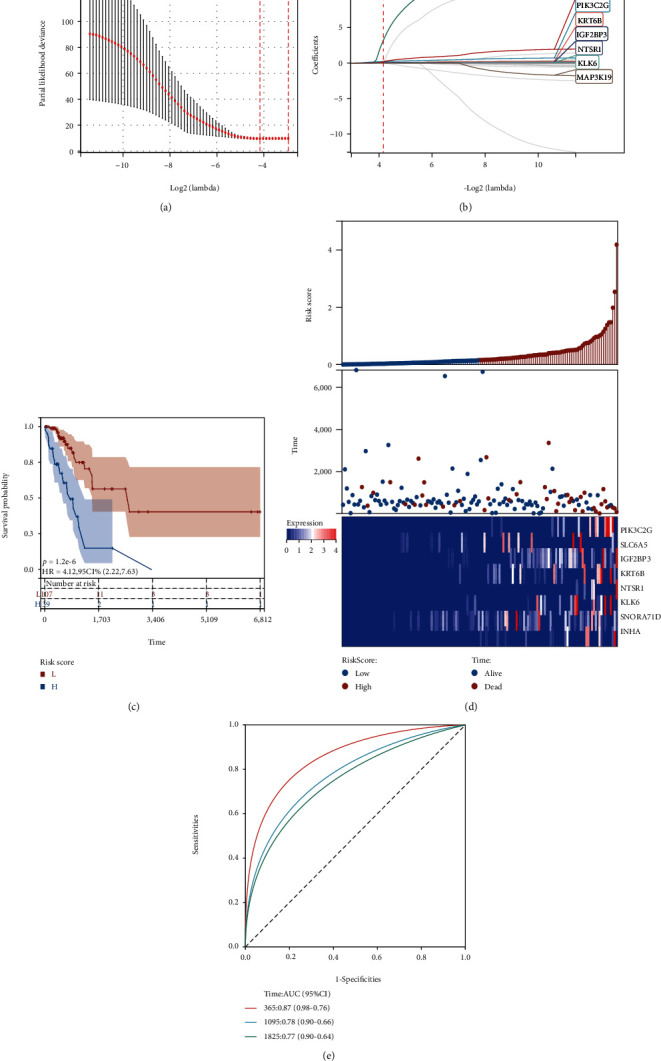
LASSO analysis of 35 differentially oxidative stress genes in the TCGA-LUAD validation set showed the distribution and prognostic analysis of 8 gene markers. (a) Cross-validation of tuning parameter selection in the LASSO model; (b) LASSO coefficient spectrum of 8 prognostic differentially oxidative stress-related genes; (c) OS-based KM survival of patients in the two groups in the TCGA-LUAD training set Curves; (d) 8 prognostic differential oxidative stress gene signature risk analysis graphs; the upper graph shows the distribution as well as the median of risk scores in the TCGA-LUAD training set; the middle graph shows the distribution of individuals in various risk groups, and the lower graph shows heatmap of differential expression of 8 genes in both risk groups; (e) AUC of time-dependent ROC curves validates the prognostic performance of risk scores in the TCGA-LUAD training set at 1, 3, and 5 years.

An 8-gene prognostic model was predicted among the genes in the validation set, including SLC6A5, SNORA71D, PIK3C2G, KRT6B, IGF2BP3, NTSR1, KLK6, and MAP3K19 as predictors of patient prognosis (Figures 6(a) and 6(b)). Both K-M survival and time-dependent ROC analysis findings were significant. The high-risk group had a greater mortality risk in comparison with the low-risk group (*p* = 1.2*e* − 6, HR = 4.12 (2.22, 7.63)) (Figure 6(c)). We show risk analysis plots for eight prognostic differential oxidative stress gene signatures, the upper panel shows the distribution and median of risk scores in the TCGA-LUAD training set, the middle panel shows the distribution of individuals in each risk group, and the lower panel shows heatmap of differential expression of 8 genes in the two risk groups (Figure 6(d)). The AUC reached 0.87 (0.98-0.76) at 1 year, 0.78 (0.90-0.66) at 3 years, and 0.77 (0.90-0.64) at 5 years (Figure 6(e)).

In the prediction model constructed based on LASSO-Cox analysis of 147 differentially oxidative stress-related genes and 35 prognostic differentially oxidative stress-related genes in the validation set, both MAP3K19 and NOS2P2 were found to be prognostic predictors, although NOS2P2 in the further multivariate Cox regression analysis results were suggested to have no statistically significant effect on prognosis. More interestingly, LASSO-Cox analysis based on 35 prognostic differentially oxidative stress-related genes found that MAP3K19 and NTSR1 could serve as prognostic predictors in both training and validation sets. Based on the above analysis, we believe that MAP3K19 and NTSR1 may have potential application value in predicting the prognosis of LUAD, and more abundant analysis is urgently needed to evaluate its application value and important clinical significance in LUAD.

### 3.6. MAP3K19 and NTSR1 Prognostic Independence of Clinical Characteristics

**Figure 7:**
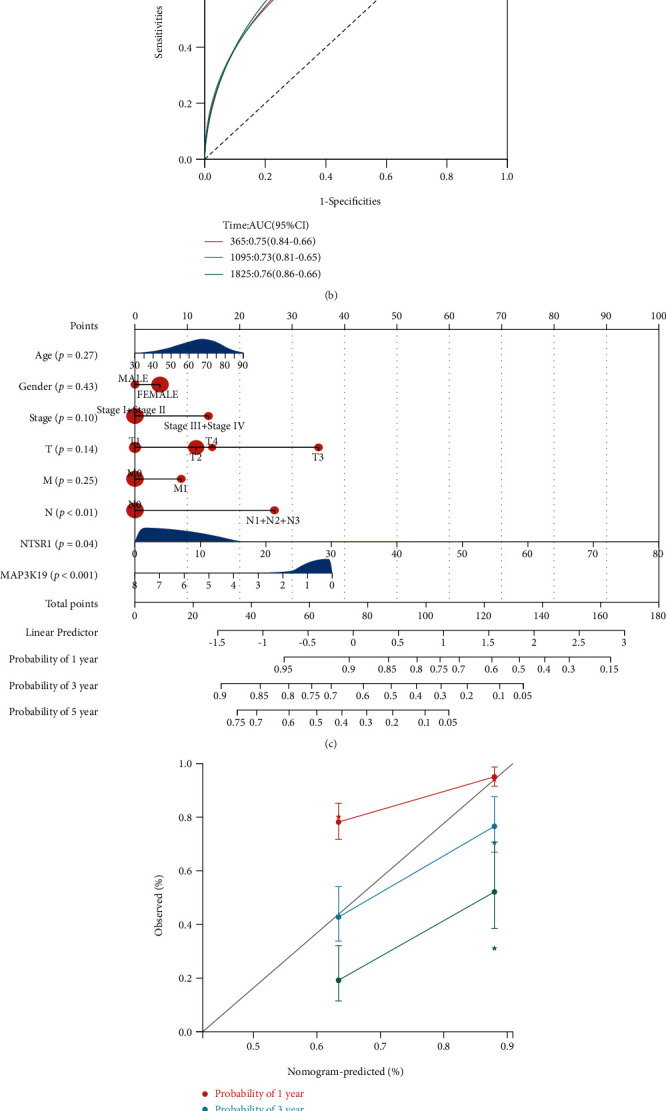
Univariate and multivariate Cox regression analyses determined the prognostic value and predictive performance of the 2-gene signature prognostic model, MAP3K19 and NTSR1. (a) K-M survival analysis revealed that the high-risk group had a higher mortality risk than the low-risk group; (b) 1, 3, and 5-year time-dependent ROC curve analysis based on OS in LUAD patients; (c) nomogram effectively integrated and shows the ability of age, sex, tumor pathological status grade, and TNM stage, as well as MAP3K19 and NTSR1 (high- and low-expression groups based on the median value) in the training set-related prognostic variables to predict LUAD overall survival; (d) calibration plot for internal validation of nomograms for overall survival prognostic status for LUAD at 1, 3, and 5 years of training set; (e) nomograms effectively integrate and demonstrate the ability of prognostic variables in TCGA-LUAD to predict overall survival in LUAD; (f) calibration plot for internal validation of nomograms for overall survival prognostic status for LUAD at 1, 3, and 5 years of the TCGA-LUAD dataset.

To further ascertain the prognostic value and predictive performance of the MAP3K19 and NTSR1 gene signatures, we first conducted a Cox regression analysis based on 445 patient samples with complete clinical information in TCGA-LUAD. We included clinical factors including age at diagnosis, gender, tumor pathological grade, lymph node status, distant metastasis, TNM stage, and gene signatures MAP3K19 and NTSR1 expression (high- and low-expression groups divided by median value). Grouped by the median RiskScore, K-M survival analysis revealed that the high-risk group had a higher mortality risk in contrast with the low-risk group (*p* = 1.1*e* − 11, HR = 3.53 (2.40, 5.19)) (Figure 7(a)). The AUC of the time-dependent ROC analysis reached 0.75 (0.84-0.66) at 1 year, 0.73 (0.81-0.65) at 3 years, and 0.76 (0.86-0.66) at 5 years (Figure 7(b)). Subsequently, we constructed nomograms to predict the prognostic status of LUAD at 1, 3, and 5 years utilizing the validation set and TCGA-LUAD overall patient samples (Figures 7(e) and 7(f)), respectively, and showed the predicted and actual nomograms with calibration plots. The nomogram effectively integrated the above prognostic variables and improved the ability to predict overall survival in LUAD. Based on the results of the validation set (Figures 7(c) and 7(d)), it was suggested that among the above prognostic factors, N (*p* < 0.01), NTSR1 (*p* = 0.04), and MAP3K19 (*p* < 0.001) were statistically significant. The above prognostic factors were validated in the TCGA-LUAD overall survival with consistent results by nomogram and calibration plot (Figures 7(e) and 7(f)).

**Figure 8:**
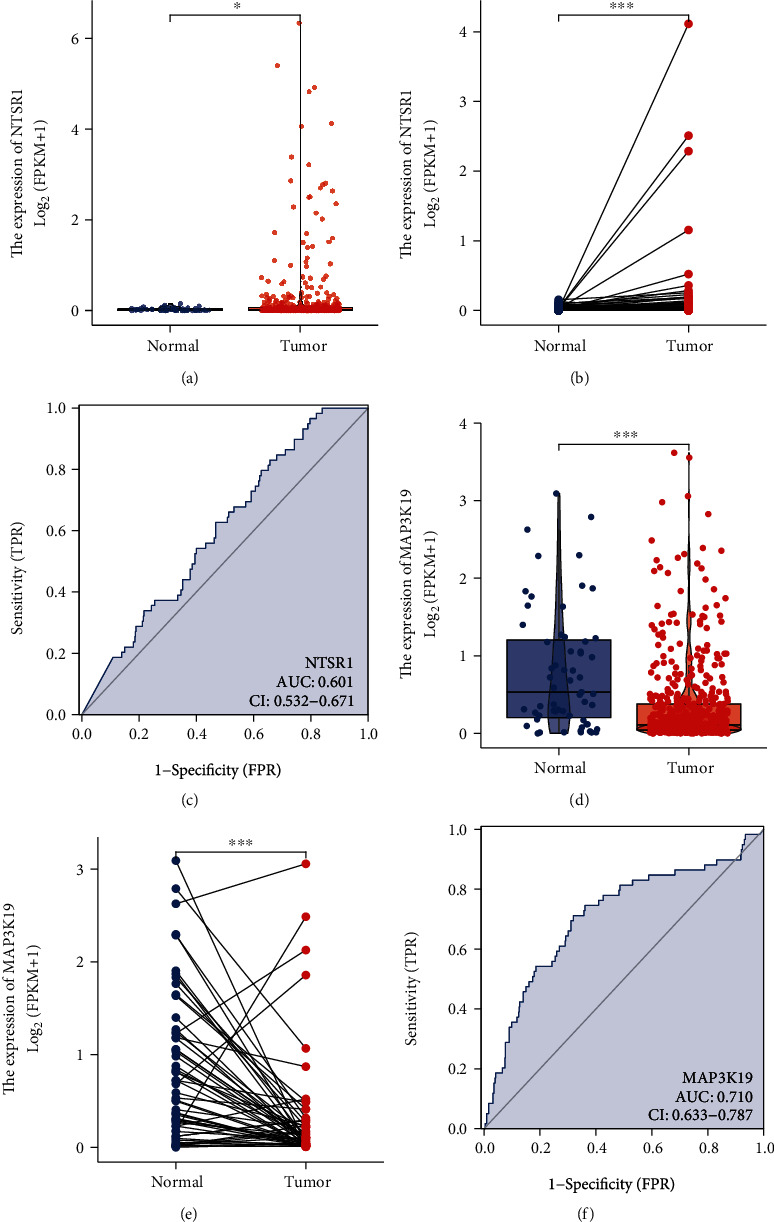
Differential expression analysis and diagnostic efficacy verification of oxidative stress-related 2-gene marker prognostic factors MAP3K19 and NTSR1 in TCGA-LUAD. (a) Unpaired differential analysis of NTSR1 in LUAD between normal and LUAD samples indicated significantly high expression; (b) paired differential analysis of NTSR1 in LUAD and normal samples suggested significantly high expression; (c) ROC curve validated MAP3K19. It has good diagnostic performance for the differential diagnosis of LUAD samples; (d) between normal and LUAD samples, the unpaired differential analysis of MAP3K19 in LUAD suggests significantly lower expression in LUAD and significantly lower expression in LUAD; (e) MAP3K19 in LUAD paired difference analysis with normal samples indicated significantly low expression; (f) ROC curve verified that MAP3K19 has good diagnostic performance for the differential diagnosis of LUAD.

The above results suggest that two gene marker prognostic factors-MAP3K19 and NTSR1 have important clinical significance for the prognosis of LUAD. Thus, we performed independent analyses for MAP3K19 and NTSR1 in TCGA-LUAD. Unpaired differential analysis between normal samples and LUAD samples indicated that NTSR1 was significantly overexpressed in LUAD (Figure 8(a)), while MAP3K19 was significantly underexpressed in LUAD (Figure 8(d)). The results of differential expression analysis of paired samples were consistent (Figures 8(b) and 8(e)). Subsequent ROC curve analysis suggested that NTSR1 (AUC: 0.601 (0.532−0.671), Figure 8(c)) and MAP3K19 (AUC: 0.710 (0.633−0.787), Figure 8(f)) had a good diagnostic performance for the differential diagnosis of LUAD samples.

**Figure 9:**
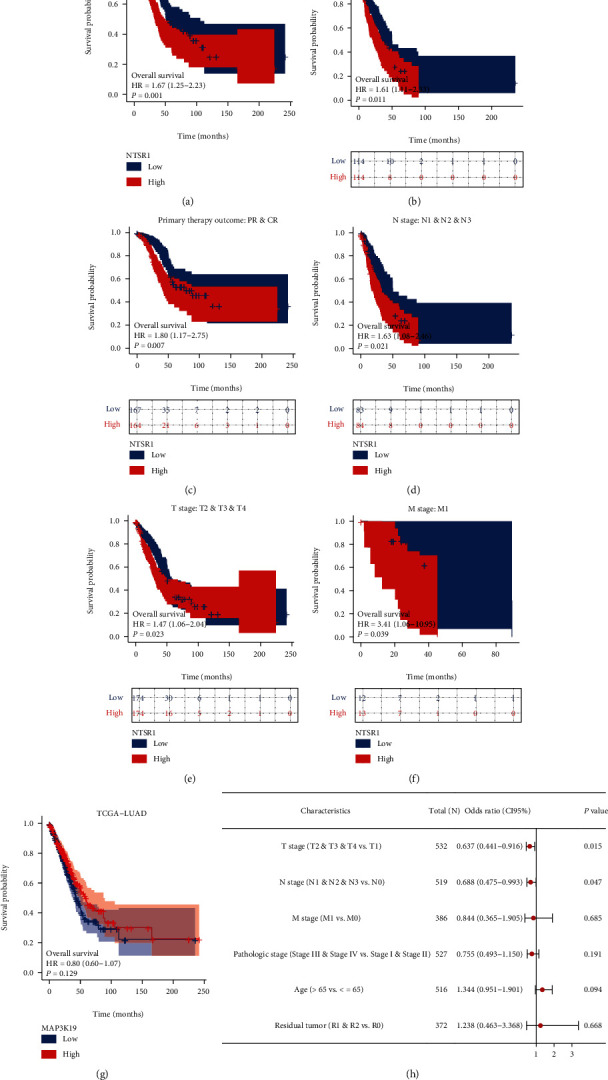
Subgroup survival analysis of clinical variables for oxidative stress-related 2 gene signatures MAP3K19 and NTSR1 in TCGA-LUAD. (a) K-M survival curves suggest that elevated NTSR1 expression level is substantially linked to poorer OS outcomes in LUAD; (b–f) K-M survival curves suggest that high NTSR1 expression is linked to poor pathologic outcomes, respectively; stage, primary therapy outcome and TNM stage were significantly correlated; (g) K-M survival curve suggested that low expression of MAP3K19 was remarkably linked to poorer OS outcome in LUAD; (h) logistic regression forest plot of binary variables of clinical subgroups of MAP3K19 showed the correlation sex.

**Figure 10:**
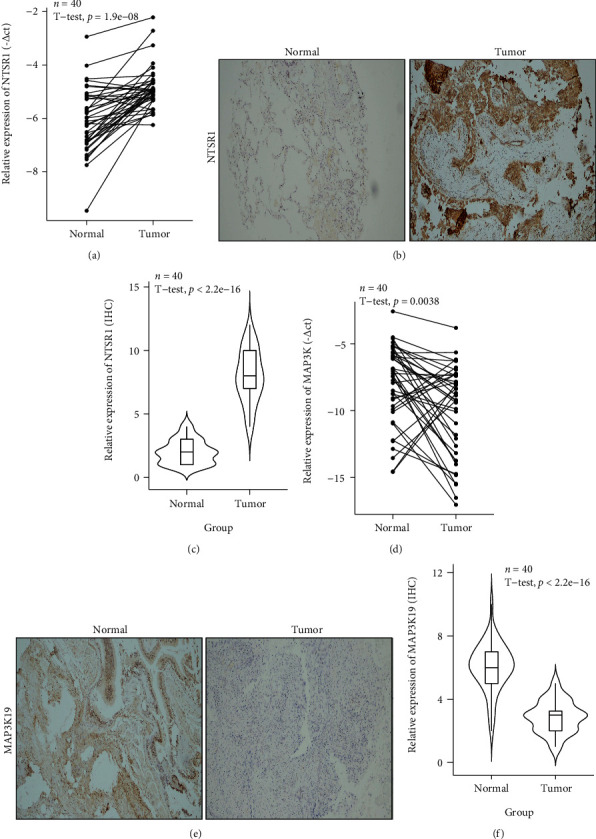
qRT-PCR and IHC analysis of model genes. (a–c) NTSR1 mRNA and protein expression were significantly increased in LUAD samples; (d–f) MAP3K19 expression in LUAD samples was significantly lower than that in paratumor normal tissue.

We further analyzed the clinical variable subgroup survival analysis of oxidative stress-related 2 gene signatures MAP3K19 and NTSR1 in TCGA-LUAD, suggesting that high NTSR1 expression and low expression of MAP3K19 were remarkably linked to poorer OS outcomes in LUAD (Figure 9(a)); subgroup survival analysis of clinical variables of primary therapy outcome, pathologic stage, and TNM stage exhibited that high expression of NTSR1 was significantly linked to poor prognosis, respectively (Figures 9(b)–9(f)). In addition, our logistic regression forest plot of dichotomous variables for clinical subgroups of MAP3K19 demonstrated correlations (not statistically significant in NTSR1 analysis) (Figure 9(h)). Meanwhile, in vivo RT-PCR and IHC results against NTSR1 (Figures 10(a)–10(c)) and MAP3K19 (Figures 10(d)–10(f)) were also consistent with our previous analysis.

### 3.7. Differential Analysis and Enrichment Analysis Based on Oxidative Stress-Related 2 Gene Signatures

All of the above analyses confirmed the superior predictive performance of MAP3K19 and NTSR1 for poor prognosis in LUAD, and the following analysis focused on exploring how the oxidative stress-related 2 gene signature might lead to poor prognosis in LUAD.

**Figure 11:**
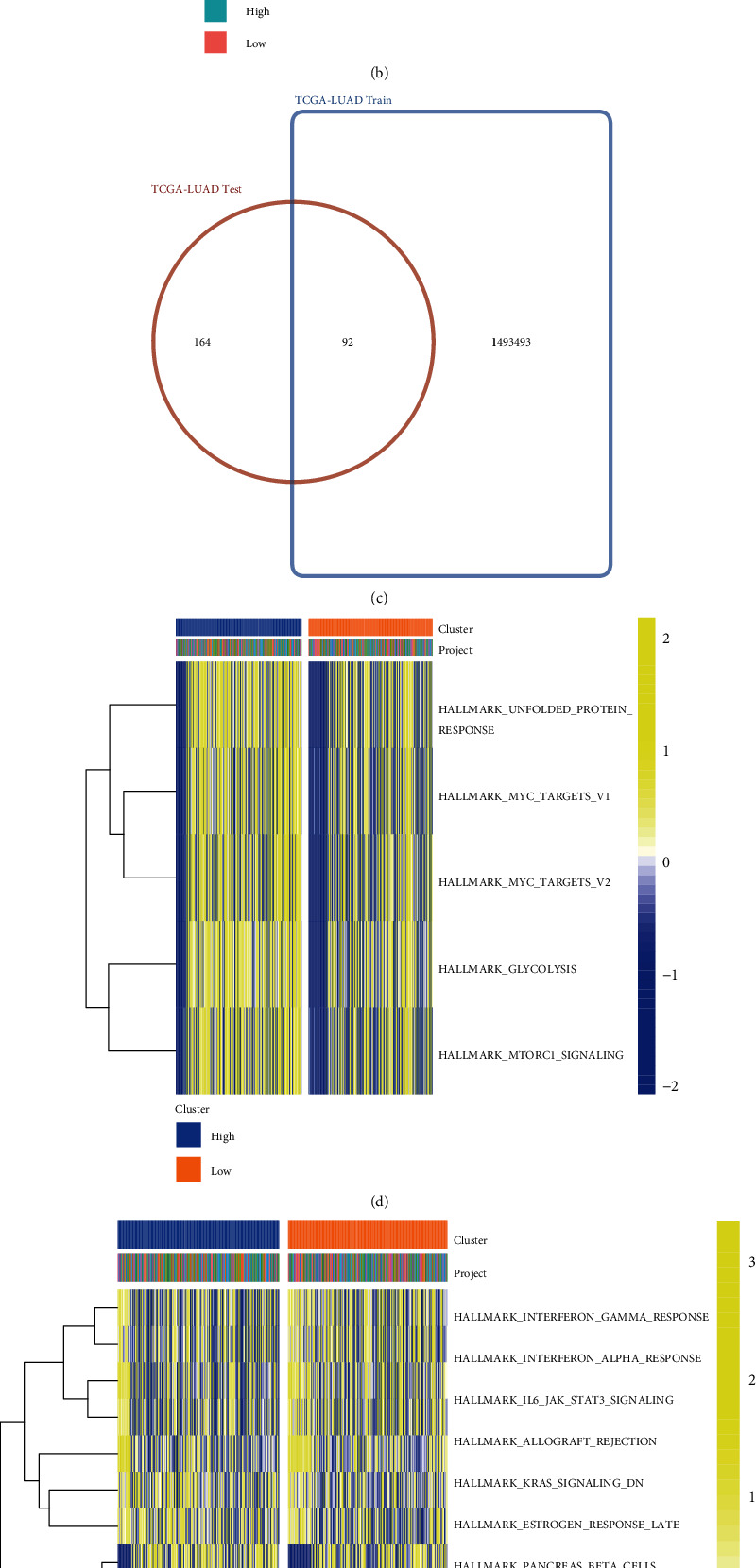
Differential analysis based on oxidative stress-related 2 gene signatures and GSVA enrichment analysis. (a, b) We obtained heatmaps of differential gene expression between the training set (a) and validation set (b) between high- and low-risk groups, correspondingly; (c) Venn diagram obtained the coexisting 92 prognostic risk genes of oxidative stress; (d–f) “h.all.v7.2.symbols.gmt” (e), “c2.cp.v7.2.symbols.gmt” with |LogFC| > 0.5 and adj.*p* < 0.05 as a heatmap (e), enriched entries for immune-related (f) gene set GSVA analysis.

In order to additionally explore the molecular mechanism engaged in the identification of high-risk populations with poor prognosis of LUAD in the oxidative stress prognostic model composed of MAP3K19 and NTSR1, we separately analyzed the significant differences between two risk groups in the prognostic model constructed by the LASSO algorithm in the training set and the validation set. We performed differential analysis on the two risk groups defined by the median risk of the training set (Figure 11(a)) and validation set (Figure 11(b)) prediction model, respectively, showing the expression of significantly different genes as a heatmap. The 92 prognostic risk genes of oxidative stress coexisting in both sets were obtained by the Venn diagram (Figure 11(c)). These genes were then subjected to enrichment analysis. GSVA analysis shows enriched entries with |LogFC| > 0.5 and adj.*p* < 0.05 as a heatmap. We then analyzed the reference gene set retrieved from the MSigDB database in “c2.cp.v7.2.symbols.gmt,” “h.all.v7.2.symbols.gmt” gene set, and immune cell infiltration-related gene set. Enrichment scores for each gene functional pathway were obtained utilizing the GSVA package in R. The heatmap visualizes the differential gene expression between the two risk groups in the training set as well as the validation set. It was found that the gene set enrichment results of “h.all.v7.2.symbols.gmt” (Figure 11(d)) and “c2.cp.v7.2.symbols.gmt” (Figure 11(e)) based on the MSigDB database were concentrated in UNFOLDED PROTEIN RESPONSE, GLYCOLYSIS, MTORC1 SIGNALING, MYC TARGETS V1, MYC TARGETS V2, and other functions and ways. Immune-related “immune.gmt” (Figure 11(f)) gene set enrichment results focused on Eosinophil, Natural.killer.cell, Immature..B.cell, Activated.B.cell, Mast.cell, and Type.1.T.helper .cell.

**Figure 12:**
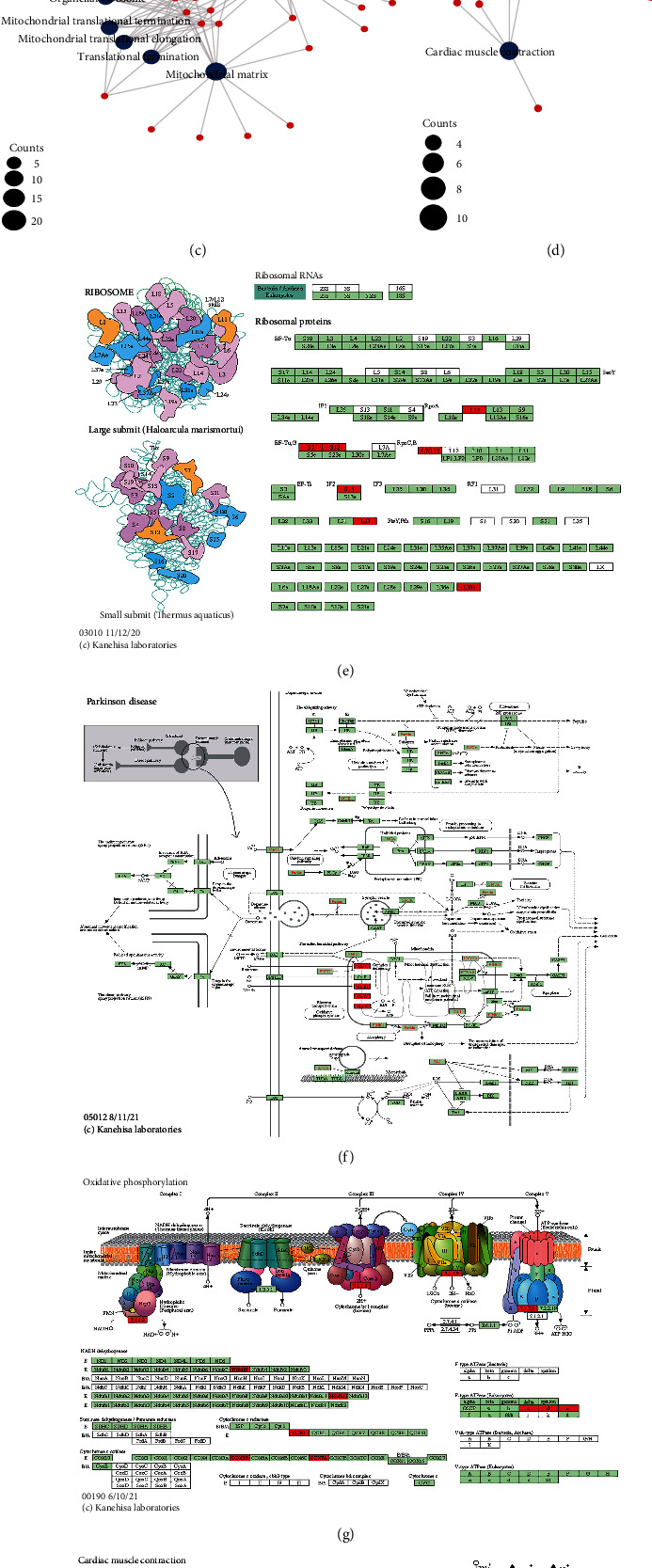
GO and KEGG enrichment analysis. (a–d) GO (a, c) and KEGG (b, d) enrichment analysis of 92 differentially expressed genes associated with LUAD prognosis. Bubble and bar graphs show the results of GO and KEGG enrichment analysis; (e–i) pathview diagrams with DEGs colored for expression are shown for hsa00190, hsa03010, hsa04260, hsa04714, and hsa05012, respectively. The figure shows term with *p*.adjust < 0.05. The length of the bars in the histogram denotes the amount of gene enrichment, the color denotes the significance, and the significance level increases gradually from blue to red.

Next, the GO (Table 3) and KEGG (Table 4) enrichment analysis results are shown by bubble plots (Figures 12(a) and 12(b)) and bar graphs (Figures 12(c) and 12(d)). BP was significantly enriched in oxidative phosphorylation, mitochondrial translational elongation, mitochondrial translational termination, translational termination, purine ribonucleoside triphosphate metabolic process, and other items. CC was significantly enriched in mitochondrial inner membrane, protein complex, matrix, ribosome, organellar ribosome, and other entries. MF was significantly enriched in the structural components of the ribosome, proton transmembrane transporter activity, cytochrome-c oxidase activity, electron transfer activity, heme-copper terminal oxidase activity, and other items. KEGG was significantly enriched in oxidative phosphorylation, thermogenesis, ribosome, Parkinson's disease, cardiac muscle contraction, and other pathways. A pathview diagram with differentially expressed genes colored for the arachidonic acid metabolism pathway is displayed in (Figures 12(e)–12(i)), including hsa00190, hsa03010, hsa04260, hsa04714, and hsa05012.

### 3.8. Gene Set Enrichment Analysis of Oxidative Stress Gene Model in LUAD

**Figure 13:**
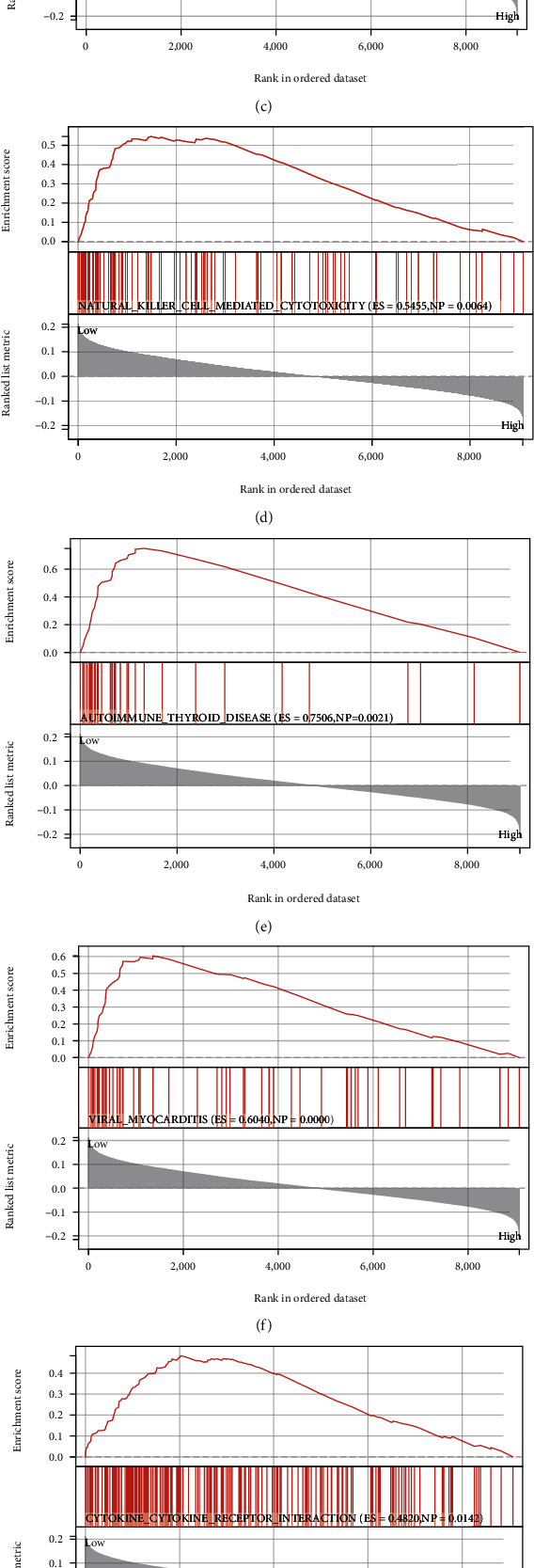
Gene set enrichment analysis of oxidative stress prognostic gene signature in LUAD. (a) JAK_STAT_SIGNALING_PATHWAY (ES = 0.5561, NP = 0.0000); (b) SYSTEMIC_LUPUS_ERYTHEMATOSUS (ES = 0.6502, NP = 0.0040); (c) LEISHMANIA_INFECTION (ES = 0.5961, NP = 0.0041); (d) NATURAL_KILLER_CELL_MEDIATED_CYTOTOXICITY (NP = 0.0064); (e) AUTOIMMUNE_THYROID_DISEASE (ES = 0.7506, NP = 0.0021); (f) VIRAL_MYOCARDITIS (ES = 0.6040, NP = 0.0000); (g) CYTOKINE_CYTOKINE_RECEPTOR_INTERACTION (ES = 0.4820, NP = 0.0142); (h) T_CELL_RECEPTOR_SIGNALING_PATHWAY (ES = 0.5250, NP = 0.0000); (i) ALLOGRAFT_REJECTION (ES = 0.7815, NP = 0.0021).

In order to additionally explore the molecular processes engaged in the identification of high-risk populations with poor prognosis of LUAD in the oxidative stress prognostic model composed of MAP3K19 and NTSR1, GSEA is a computational method utilized to ascertain if a predefined set of genes shows statistical differences between two biological states, which is typically utilized to estimate expression in a dataset sample. Changes in the pathway as well as biological process activity were analyzed [23]. To investigate the variations in biological mechanisms between the two groups of samples, employing the gene expression profile dataset, we obtained the reference gene set “c2.cp.kegg.v7.4.entrez.gmt” from the MSigDB database, utilizing the R package “clusterProfiler.” The GSEA method is included in enrichment analysis and visualization of datasets. *p* value < 0.05 was considered statistically significant (Figure 13 and Table 5).

### 3.9. Pan-Cancer Analysis of Immune Cells and Immune Infiltration Based on MAP3K19 and NTSR1

**Figure 14:**
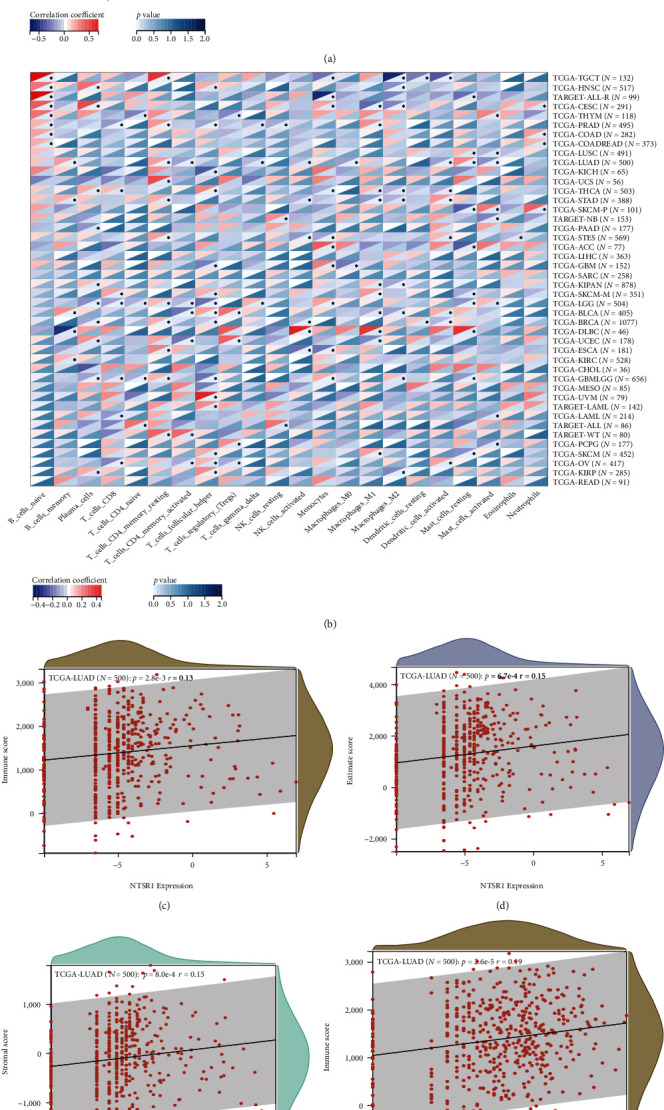
Immune cell infiltration analysis. (a, b) CIBERSORT analysis of MAP3K19 (a) and NTSR1 (b) heatmaps related to the infiltration abundance of immune cells in pan-cancer; (c–h) ESTIMATE analyzed the scores of tumor purity, stromal cell level, and immune cell infiltration level in tumor tissue and showed NTSR1 (c–e) and MAP3K19 (f–h) expression with immune score, ESTIMATE score, and stromal score, respectively, with a scatterplot positively correlated.

The infiltration abundance of immune cells was analyzed by CIBERSORT, and the correlation heatmap in pan-cancer showed that MAP3K19 (Figure 14(a)) and NTSR1 (Figure 14(b)) were significantly correlated with immune infiltration in more types of tumors. Based on TCGA LUAD transcription profile and CIBERSORT, we derived the proportions of 22 tumor-infiltrating immune cells. In our study, the use of RNA-seq data as well as global proteomic data inferred MAP3K19 and NTSR1 for pan-cancer tumor purity, immune score, and stromal score. From this, significantly correlated immune infiltration scores were identified, and we analyzed the scores for stromal cell levels, tumor purity, and immune cell infiltration levels in cancer tissues calculated with ESTIMATE expression was substantially positively linked to immune score, ESTIMATE score, and stromal score, respectively. The results suggest that NTSR1 (Figures 14(c)–14(e)) and MAP3K19 (Figures 14(f)–14(h)) were closely related to stromal cell level, tumor purity, and immune cell infiltration level in the lung tumor microenvironment and may affected the prognosis of LUAD by changing the tumor immune microenvironment.

### 3.10. Correlation Analysis of Immune Cell Infiltration

**Figure 15:**
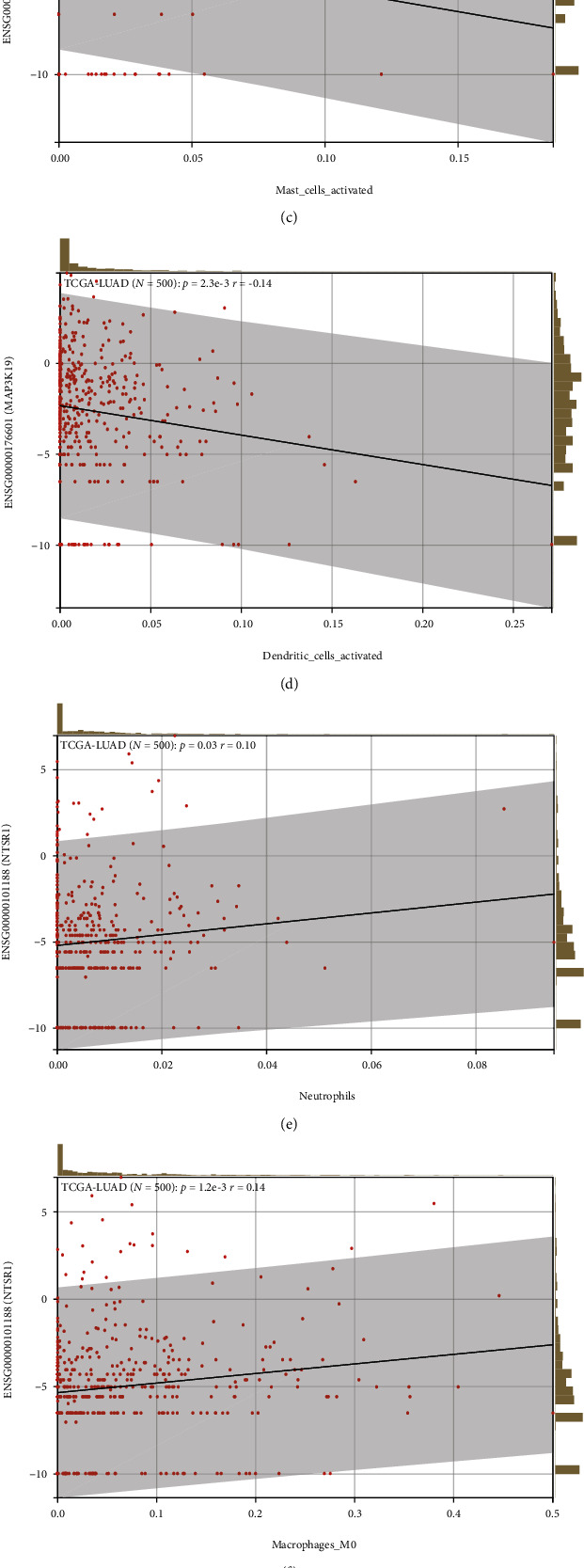
Correlation analysis of immune cell infiltration. (a, b) MAP3K19 was positively correlated with T cells CD4 memory resting and B cells memory; (c, d) MAP3K19 was negatively correlated with mast cells activated and dendritic cells activated; (e–g) NTSR1 was positively correlated with neutrophils, macrophages M0, and T cells gamma delta; (h) NTSR1 was negatively linked to mast cells resting.

It was found that MAP3K19 was positively correlated with T cell CD4 memory resting and B cell memory (Figures 15(a) and 15(b)) and negatively correlated with mast cells activated and dendritic cells activated (Figures 15(c) and 15(d)). NTSR1 was positively correlated with neutrophils, macrophages M0, and T cell gamma delta (Figures 15(e)–15(g)) and negatively linked to mast cells resting (Figure 15(h)). In our study, the use of RNA-seq data as well as global proteomic data inferred MAP3K19 and NTSR1 for pan-cancer tumor purity, immune score, and stromal score. From this, significantly correlated immune infiltration scores were identified, and we analyzed the scores for stromal cell levels, tumor purity, and immune cell infiltration levels in cancer tissues calculated with ESTIMATE expression which was substantially positively linked to immune score, ESTIMATE score, and stromal score, respectively. The results suggest that NTSR1 (Figures 14(c)–14(e)) and MAP3K19 (Figures 14(f)–14(h)) were closely related to stromal cell level, tumor purity, and immune cell infiltration level in the lung tumor microenvironment and may affected the prognosis of LUAD by changing the tumor immune microenvironment.

## 4. Discussion

Globally, lung cancer is the major cause of cancer-related mortalities, whereas LUAD is the most prevalent histological subtype of the disease. About two-thirds of LUAD have activated oncogenes. Most oncogene mutations often activate downstream signaling pathways through oxidative stress pathways and states, ultimately leading to lethal malignancies, including LUAD [24, 29, 30]. Molecularly targeted therapy significantly improves survival in patients with treatment-targeted lesions compared to conventional chemotherapy [31, 32]. However, the clinical efficacy of targeted drugs has been improved due to the lack of appropriate small molecules to bind major tumor-causing gene mutations.

For the survival of both normal cells and cancerous cells, redox hemostasis is fundamental. Nonetheless, numerous malignancies have increased levels of reactive oxygen species (ROS) and exhibit signs of chronic oxidative stress as a result of oncogenic injury, metabolic malformations, hypoxia, and proteotoxic stress [33]. The increased ROS at the sublethal level is implicated in the enhancement of tumor development via triggering mutations and altering cell signaling [34]. Because traditional cytotoxic agents additionally influence normal tissues, targeted approaches that induce catastrophic oxidative stress selectively in malignant cells would avail a better therapeutic window [35].

The LUAD cohort of TCGA availed both the expression and clinical data in this study. Among oxidative stress-related genes, we did differential expression analysis as well as univariate Cox analysis to screen 32 prognostic DEGs from 147 differentially expressed oxidative stress-related genes and utilized Lasso-penalized Cox regression analysis constructed 2 gene markers associated with prognosis. At the same time, by constructing a PPI network, we analyzed the distribution of 18 differentially oxidative stress hub genes in transport vesicles, endoplasmic reticulum lumen, etc., affecting blood pressure, hormone secretion, nucleotide second messengers, and other functions to regulate oxidative stress effect. To construct and validate the oxidative stress gene signature affecting the diagnosis and prognosis of LUAD, we randomly divided 445 patients with complete clinical informatics data in TCGA-LUAD into the training set (2/3 of the total, *n* = 296) and the test set (1/3 of the total, *n* = 149); the grouping is normally distributed. We first performed a univariate Cox regression analysis of gene expression premised on the expression data of 147 oxidative stress-related genes in the training and test set, correspondingly. Thirty-five genes with prognostic values coexisting in both sets were identified. Then, we performed LASSO-Cox proportional hazards regression based on 35 prognostic-related differential oxidative stress genes in the training and validation set, correspondingly, and found that MAP3K19 and NTSR1 in both sets showed a better prediction of poor prognosis in LUAD ability. Therefore, the prognostic independence of 2-gene signature prognostic factors, MAP3K19 and NTSR1, was further analyzed in combination with clinical features. A nomogram was constructed to effectively integrate prognostic variables and validated with calibration. Next, the difference analysis of MAP3K19 and NTSR1 in TCGA-LUAD and the correlation analysis of clinical variables indicated that both the high expression of NTSR1 and the low expression of MAP3K19 had a better diagnostic performance for the diagnosis of LUAD and the identification of poor prognosis. At the same time, MAP3K19 and NTSR1 were found to be significantly correlated with clinical variables such as LUAD pathological stage and TNM grade. Prognosis-related differential oxidative stress gene signatures are independent prognostic factors in patients with LUAD.

The importance of tumor immune activity on tumorigenesis and development, as well as individual variation at the gene level, has attracted more and more researchers to focus on the significance of differential genes that may be useful in distinguishing pRCC patients with heterogeneous responses and predicting prognosis potentially meaningful. The ESTIMATE analysis of pan-cancer species analysis of MAP3K19 and NTSR1 in TCGA database systematically recorded the abundance of 22 tumor-infiltrating immune compartments in LUAD samples through the CIBERSORT algorithm and integrated it with the MAP3K19 and NTSR1 molecular profiles to analyze the degree of immune infiltration in LUAD. Subsequently, further immune score, stromal score, and ESTIMATE score in the pan-cancer TME revealed the potential roles of MAP3K19 and NTSR1 in regulating stromal/immune scores and gene expression in tumors. This suggests that the oxidative stress prognostic model composed of MAP3K19 and NTSR1 may be involved in the molecular mechanism in the identification of high-risk populations with poor prognosis of LUAD.

Studies have shown that MAP3K19 level is elevated in COPD and bronchoalveolar lavage macrophages from patients with IPF. At the level of target gene transcription or protein synthesis, molecular studies have confirmed that MAP3K19 inhibitors are linked to pirfenidone or nintedanib. At the same time, MAP3K19 significantly attenuated bleomycin-induced pulmonary fibrosis [36] and is a central mediator of cigarette smoke-induced pulmonary inflammation and lower airway destruction [37]. In studies on lung cancer, targeting MAP3K19 has been reported to prevent human lung myofibroblast activation in vitro and in a humanized SCID model of idiopathic pulmonary fibrosis [38]. On the other hand, it can also phosphorylate MAP2K, thereby activating ERK as well as JNK kinases and increasing the KRAS mutant lung cancer cells' viability [39]. The mechanism of action of NTSR1 in various tumors has also been reported many times [40]. Effects, underlying mechanisms, and clinical roles of NTSR1 on gastric adenocarcinoma cell proliferation and invasion. Interfering with NTSR1 expression exhibits anti-invasive effects through the Jun/miR-494/SOCS6 axis in glioblastoma cells [41]. The mechanism of action of NTSR1 in various tumors has also been reported many times. Effects, underlying mechanisms, and clinical roles of NTSR1 on gastric adenocarcinoma cell proliferation and invasion. Interfering with NTSR1 expression causes anti-invasive effects through the Jun/miR-494/SOCS6 axis in glioblastoma cells. NTSR1 and Wnt/*β*-catenin enhance tumor growth in glioblastoma [42]. Furthermore, NTSR1 methylation is linked to the lateral and noninvasive progression of colorectal tumors, whereas lowered levels of methylation might enhance the malignant potential via activation of NTSR1 [43].

In conclusion, this study constructed and validated a 2-gene oxidative stress-related LUAD prognostic model, MAP3K19 and NTSR1, which were significantly correlated with clinical variables (including LUAD pathological stage and TNM grade) and significantly affected the infiltration of immune cells in the tumor microenvironment (TME) of LUAD. And they are all involved in the process of oxidative stress and the energy metabolism network of ROS. The results of enrichment analysis based on the biological functions of GO, KEGG, and GSEA and pathway signaling patterns suggest that the molecular expression of MAP3K19 and NTSR1 and other immune cells help in the process of oxidative stress, even though conclusive evidence is still unavailable. Studies have shown that during apoptosis, immune cells are attracted and aggregated by a set of signals that enhance programmed cell death [44, 45]. In terms of bioinformatics, various studies have revealed a possible link between tumor and immune infiltration [46, 47]. Additionally, to palliative targeted therapy, monotherapy with new immunotherapies, such as immune checkpoint inhibitors (ICIs), has also demonstrated quite successful outcomes in some individuals with advanced LUAD [48]. In this study, through risk group-based immune annotation analysis, we found that macrophages, Tregs, and other types of immune cells and costimulation of immune-related roles were significantly enriched in both cohorts, suggesting that there may be potential regulatory mechanisms.

This study found some limitations. In a bioinformatics study, the weakness of the absence of experimental as well as clinical validation remains, and the utilization of alternative cutoff criteria, statistical methodologies, or analytical tools may provide varied results. Furthermore, building a prognostic model by focusing on a single marker may result in the deletion of numerous other potential prognostic genes. In conclusion, we created a novel 2-gene signature associated with oxidative stress that was shown to be an independent prognostic predictor of OS in LUAD. Through functional annotation analysis, the gene signature was associated with tumor immunity; nonetheless, its underlying process is not clear and needs to be explored further.

The TCGA-LUAD cohort availed both the clinical data and the expression data in this study. Among oxidative stress-related genes, we did differential expression analysis and univariate Cox analysis in order to screen 32 prognostic DEGs from 147 differentially expressed oxidative stress-related genes and utilized Lasso-penalized Cox regression analysis constructed 2 gene markers associated with prognosis. At the same time, by constructing a PPI network, we analyzed the distribution of 18 differentially oxidative stress hub genes in transport vesicles, endoplasmic reticulum lumen, etc., affecting blood pressure, hormone secretion, nucleotide second messengers, and other functions to regulate oxidative stress effect. To construct and validate the oxidative stress gene signature affecting the diagnosis and prognosis of LUAD, we randomly divided 445 patients with complete clinical informatics data in TCGA-LUAD into the training set (2/3 of the total, *n* = 296) and the test set (1/3 of the total, *n* = 149); the grouping is normally distributed. We first performed a univariate Cox regression analysis of gene expression premised on the expression data of 147 oxidative stress-related genes in the training and test sets, respectively. Thirty-five genes with prognostic values coexisting in both sets were identified. Then, we performed LASSO-Cox proportional hazards regression based on 35 prognostic-related differential oxidative stress genes in the training set as well as the validation set, respectively, and found that MAP3K19 and NTSR1 in the two sets showed a better prediction of poor prognosis in LUAD ability. Therefore, the prognostic independence of 2-gene signature prognostic factors, MAP3K19 and NTSR1, was further analyzed in combination with clinical features. A nomogram was constructed to effectively integrate prognostic variables and validated with calibration. Next, the difference analysis of MAP3K19 and NTSR1 in TCGA-LUAD and the correlation analysis of clinical variables indicated that both the high expression of NTSR1 and the low expression of MAP3K19 had a better diagnostic performance for the diagnosis of LUAD and the identification of poor prognosis. At the same time, MAP3K19 and NTSR1 were found to be significantly correlated with clinical variables such as LUAD pathological stage and TNM grade. Prognosis-related differential oxidative stress gene signatures are independent prognostic factors in patients with LUAD. ESTIMATE analysis results of pan-cancer species analysis of MAP3K19 and NTSR1 in TCGA database. The abundances of 22 tumor-infiltrating immune compartments in LUAD samples were systematically recorded by the CIBERSORT algorithm and integrated with MAP3K19 and NTSR1 molecular profiles to analyze the degree of immune infiltration in LUAD. Subsequently, further immune score, stromal score, and ESTIMATE score in the pan-cancer TME revealed the potential roles of MAP3K19 and NTSR1 in regulating stromal/immune scores and gene expression in tumors. This suggests that the oxidative stress prognostic model composed of MAP3K19 and NTSR1 may be involved in the molecular mechanism in the identification of high-risk populations with poor prognosis of LUAD.

## Figures and Tables

**Table 1:** Clinical characteristics of patients in TCGA-LUAD internal training set and validation set.

Characteristics	Train (*N* = 296)	Test (*N* = 149)	Total (*N* = 445)	*p* value	FDR
Age					
Mean ± SD	64.86 ± 10.00	65.05 ± 10.21	64.93 ± 10.06		
Median [min-max]	66.00 [33.00, 88.00]	66.00 [41.00, 86.00]	66.00 [33.00, 88.00]		
Gender				1	1
Female	162 (36.40%)	81 (18.20%)	243 (54.61%)		
Male	134 (30.11%)	68 (15.28%)	202 (45.39%)		
Stage				0.43	1
Stage I	157 (35.28%)	87 (19.55%)	244 (54.83%)		
Stage II	68 (15.28%)	36 (8.09%)	104 (23.37%)		
Stage III	54 (12.13%)	21 (4.72%)	75 (16.85%)		
Stage IV	17 (3.82%)	5 (1.12%)	22 (4.94%)		
T				0.88	1
T1	108 (24.27%)	49 (11.01%)	157 (35.28%)		
T2	152 (34.16%)	80 (17.98%)	232 (52.13%)		
T3	24 (5.39%)	14 (3.15%)	38 (8.54%)		
T4	12 (2.70%)	6 (1.35%)	18 (4.04%)		
M				0.52	1
M0	198 (44.49%)	100 (22.47%)	298 (66.97%)		
M1	17 (3.82%)	5 (1.12%)	22 (4.94%)		
MX	81 (18.20%)	44 (9.89%)	125 (28.09%)		
N				0.19	0.93
N0	186 (41.80%)	105 (23.60%)	291 (65.39%)		
N1	54 (12.13%)	27 (6.07%)	81 (18.20%)		
N2	49 (11.01%)	17 (3.82%)	66 (14.83%)		
N3	2 (0.45%)	0 (0.0*e*+0%)	2 (0.45%)		
NX	5 (1.12%)	0 (0.0*e*+0%)	5 (1.12%)		

**Table 2:** List of GO and KEGG enrichment analysis results of differential oxidative stress hub genes with close interaction.

Ontology	ID	Description	*p* value	*p*.adjust	*Q* value
BP	GO:0008217	Regulation of blood pressure	4.92*e*-05	0.008	0.004
	GO:0090276	Regulation of peptide hormone secretion	7.32*e*-05	0.008	0.004
	GO:0030072	Peptide hormone secretion	1.26*e*-04	0.008	0.004
	GO:0007187	G protein-coupled receptor signaling pathway, coupled to cyclic nucleotide second messenger	1.36*e*-04	0.008	0.004
	GO:0046883	Regulation of hormone secretion	1.52*e*-04	0.008	0.004

CC	GO:0030658	Transport vesicle membrane	0.003	0.039	0.024
	GO:0005788	Endoplasmic reticulum lumen	0.006	0.039	0.024
	GO:0031045	Dense core granule	0.008	0.039	0.024
	GO:0042629	Mast cell granule	0.009	0.039	0.024
	GO:0030133	Transport vesicle	0.010	0.039	0.024

MF	GO:0048018	Receptor ligand activity	6.48*e*-04	0.014	0.008
	GO:0005179	Hormone activity	9.68*e*-04	0.014	0.008
	GO:0005184	Neuropeptide hormone activity	0.011	0.067	0.036
	GO:0071855	Neuropeptide receptor binding	0.013	0.067	0.036
	GO:0042056	Chemoattractant activity	0.015	0.067	0.036

KEGG	hsa04923	Regulation of lipolysis in adipocytes	4.83*e*-04	0.005	0.001
	hsa04080	Neuroactive ligand-receptor interaction	7.00*e*-04	0.005	0.001
	hsa04024	cAMP signaling pathway	0.007	0.032	0.009
	hsa04950	Maturity onset diabetes of the young	0.016	0.056	0.017
	hsa04913	Ovarian steroidogenesis	0.031	0.064	0.019

**Table 3:** GO enrichment analysis of coexpressed genes based on the LUAD oxidative stress-related prognostic risk model.

Ontology	ID	Description	BgRatio	*p* value	*p*.adjust	*Q* value
BP	GO:0006119	Oxidative phosphorylation	145/18670	8.76*e*-10	1.28*e*-06	1.17*e*-06
	GO:0070125	Mitochondrial translational elongation	88/18670	5.21*e*-09	2.78*e*-06	2.53*e*-06
	GO:0070126	Mitochondrial translational termination	89/18670	5.70*e*-09	2.78*e*-06	2.53*e*-06
	GO:0006415	Translational termination	104/18670	1.97*e*-08	7.19*e*-06	6.56*e*-06
	GO:0009205	Purine ribonucleoside triphosphate metabolic process	335/18670	2.83*e*-08	7.42*e*-06	6.76*e*-06

CC	GO:0005743	Mitochondrial inner membrane	473/19717	3.79*e*-18	7.50*e*-16	6.10*e*-16
	GO:0098798	Mitochondrial protein complex	262/19717	1.10*e*-10	1.09*e*-08	8.85*e*-09
	GO:0005759	Mitochondrial matrix	469/19717	1.45*e*-08	9.60*e*-07	7.81*e*-07
	GO:0000313	Organellar ribosome	87/19717	1.03*e*-07	4.09*e*-06	3.33*e*-06
	GO:0005761	Mitochondrial ribosome	87/19717	1.03*e*-07	4.09*e*-06	3.33*e*-06

MF	GO:0003735	Structural constituent of ribosome	202/17697	3.60*e*-06	9.07*e*-04	7.84*e*-04
	GO:0015078	Proton transmembrane transporter activity	133/17697	3.07*e*-05	0.004	0.003
	GO:0009055	Electron transfer activity	114/17697	1.66*e*-04	0.011	0.009
	GO:0004129	Cytochrome-c oxidase activity	28/17697	2.69*e*-04	0.011	0.009
	GO:0015002	Heme-copper terminal oxidase activity	28/17697	2.69*e*-04	0.011	0.009

**Table 4:** KEGG enrichment analysis of coexpressed genes based on the LUAD oxidative stress-related prognostic risk model.

Ontology	ID	Description	BgRatio	*p* value	*p*.adjust	*Q* value
KEGG	hsa00190	Oxidative phosphorylation	133/8076	4.47*e*-08	3.35*e*-06	2.82*e*-06
	hsa04714	Thermogenesis	231/8076	4.98*e*-07	1.87*e*-05	1.57*e*-05
	hsa03010	Ribosome	158/8076	2.71*e*-05	6.77*e*-04	5.70*e*-04
	hsa05012	Parkinson disease	249/8076	6.90*e*-05	0.001	0.001
	hsa04260	Cardiac muscle contraction	87/8076	1.25*e*-04	0.002	0.001

**Table 5:** Results of gene set enrichment analysis of gene signatures in prognostic models of oxidative stress.

Term	ES	NES	*p* value	FDR
VIRAL_MYOCARDITIS	0.604	2.0023	0.0117	0.04
AUTOIMMUNE_THYROID_DISEASE	0.7506	2.0069	0.0021	0.0149
ASTHMA	0.7919	2.0269	0.002	0.0168
NATURAL_KILLER_CELL_MEDIATED_CYTOTOXICITY	0.5455	1.9577	0.0064	0.0191
JAK_STAT_SIGNALING_PATHWAY	0.5561	2.0488	0.0259	0.023
LEISHMANIA_INFECTION	0.5961	1.8795	0.0041	0.0266
ALLOGRAFT_REJECTION	0.7815	1.9236	0.0021	0.0269
T_CELL_RECEPTOR_SIGNALING_PATHWAY	0.525	1.8848	0.0288	0.0149
SYSTEMIC_LUPUS_ERYTHEMATOSUS	0.6502	1.897	0.004	0.0293
CYTOKINE_CYTOKINE_RECEPTOR_INTERACTION	0.482	1.8142	0.0142	0.046
TOLL_LIKE_RECEPTOR_SIGNALING_PATHWAY	0.4956	1.8005	0.0151	0.0487

## Data Availability

The datasets used and/or analyzed during the current study are available from the corresponding author on reasonable request.
